# The Impact of Health Care Funding on Interprofessional Collaboration and Integrated Service Delivery in Primary and Allied Care: Protocol for a Scoping Review

**DOI:** 10.2196/36448

**Published:** 2022-05-13

**Authors:** Jessica Archer, Luke Robinson, Ted Brown

**Affiliations:** 1 Department of Occupational Therapy Faculty of Medicine, Nursing, and Health Sciences Monash University Frankston Australia

**Keywords:** allied health, healthcare funding, interprofessional collaboration, integrated healthcare, primary health, primary care

## Abstract

**Background:**

Improving funding models and implementing policies that facilitate greater interprofessional collaboration and integration at the primary and allied health level could improve the ongoing quality and safety and future sustainability of the wider health care system by reducing inefficiencies and inequalities. Defining these key health care funding–related models, policies, and concepts, identifying research gaps, and systematically mapping the associated literature will inform future research on this topic.

**Objective:**

The aim of this scoping review is to provide a descriptive overview of contemporary health care funding models and the key policies involved in the delivery of primary and allied health care. Further, it will investigate the impact these models and policies have on interprofessional collaboration and integrated service delivery at the primary and allied health care levels.

**Methods:**

A search of published and grey literature will be conducted using the following databases: the Allied and Complementary Medicine Database, CINAHL, Embase, Emcare, MEDLINE, PsycINFO, Scopus, Open Access Theses and Dissertations, and Web of Science. The search will be limited to resources available in the English language and published since 2011. Following the search, an independent screening of titles and abstracts will be undertaken by 2 independent reviewers, with a third reviewer available to resolve any potential disagreements. Full-text resources will then be assessed against the inclusion criteria following the same process. Extracted data will be presented using a convergent narrative approach, accompanied by tables and figures.

**Results:**

Electronic database searches have retrieved 8013 articles. The results of this scoping review are expected in May 2022.

**Conclusions:**

The findings from this review will be used to inform future research projects investigating the role of primary health care funding, interprofessional collaboration, and service integration in improving health care access, efficiency, effectiveness, and sustainability.

**International Registered Report Identifier (IRRID):**

DERR1-10.2196/36448

## Introduction

### Background

According to the World Health Organization (WHO), to meet current and predicted future global health care needs, governments and health care funding bodies worldwide must prioritize health care funding models and policies that embrace universal health coverage (UHC) [1-4]. Health systems designed and funded to reflect the principles of UHC ensure that everyone who needs access to health care resources can access the services they need at a time and place that is suitable and without experiencing financial hardship [1-4]. In general, health systems considered to be funded effectively do not necessarily spend the most money or account for the highest percentage of gross domestic product per capita [5,6]. Rather, more efficient, and strategic health care spending is directed toward systems favoring funding models and policies that value team care approaches and health care professionals moving away from traditionally fragmented professional practice silos where disciplines are largely segregated [7-12]. In this context, instead of health care professionals working as specialists in relative isolation, providers from different professional backgrounds are encouraged to work together through interprofessional collaboration [7-12].

Interprofessional collaboration, by definition, uses the collective knowledge and expertise of 2 or more health care professions working together to offer more appropriate, timely, comprehensive, and person-centered health care to consumers [2,6,8-12]. In health care systems with effective funding models and policies, interprofessional collaborations are integrated horizontally across different health care professions and vertically between each level of the health care system [8,9]. This integration is inclusive of tertiary (eg, hospitals), secondary (eg, specialist health services such as psychiatry, oncology, and surgery), and primary (eg, general and community health services, such as general practice, community health, and allied health), levels of care [8,9]. As such, service integration connects the different levels and disciplines involved in the health care system with administrative and organizational support [8,9]. In turn, this can improve clinical outcomes, increase consumer and clinician satisfaction with service provision, and promote more efficient use of health care resources, staff, and funding [8,9].

Since 1978’s Declaration of Alma-Ata, which was more recently renewed and ratified with 2018’s Declaration of Astana, the WHO has strongly endorsed commitments to increase health care funding and improve funding models and policies at the primary level [1,8,9,13-15]. A growing body of evidence suggests that concentrating on primary health and structuring future health systems around strong primary foundations will have the greatest impact on efforts to move toward UHC [1,8,9,13-15]. Focusing on funding improvements at this level is understood to have the most significant impact as the primary level is the largest, most professionally diverse, and most geographically spread level of care and is responsive to the determinants of both health and ill-health [1,2]. These features also suggest that primary health is the most naturally accommodating and well-suited to interprofessional collaboration and integrated service provision [8,9,16,17]. Likewise, they contribute to primary health by being uniquely positioned to assist the largest numbers of people and to do so earlier during illness or injury progression [1,2,6]. As such, a strong primary health care system can delay, mitigate, and in some cases even prevent the need to escalate service provision to more specialized, centrally located, scarce, and more costly secondary and tertiary health care services [1,2,6,8].

However, despite many varied and concerted international efforts to commit to UHC goals and improve primary health care funding models and policies [2,4,6], primary health clinicians around the world are still working in underfunded vertically and horizontally isolated professional practice silos [7-12]. Funding policies and models are also ambiguous, creating many barriers to interprofessional collaboration and integrated health care delivery [2,6,15,18,19].

Since the beginning of the COVID-19 pandemic, existing health care inequities embedded within contemporary funding models and policies have become increasingly apparent [3]. In some cases, the divide between already marginalized groups has significantly worsened, putting extra pressures on health care systems to limit who, how, where, and when consumers can access health care services as well as the financial cost for doing so [3]. This increasing divide in health care equity is also occurring in the context of depleted financial reserves, stretched and interrupted global resource and supply chains, and current and predicted future health care staffing shortages [3].

Although these issues present substantial challenges to global economic and health systems, they also offer governments a unique window of opportunity to rethink health care funding models and policies. By reorienting health systems to better reflect the fundamental values of UHC as part of the pandemic recovery process, there is potential to instill greater ongoing financial and workforce sustainability [3]. However, to maximize improvements to health care efficiency and access, more research is needed to determine if and how health care funding policies and models might impact interprofessional collaboration and service integration, starting at the primary health care level.

A preliminary search of MEDLINE, CINAHL, Open Science Framework, and JMIR Research Protocols identified no current or in-progress reviews focusing on the role of health care funding in relation to interprofessional collaboration or integrated health care. Instead, previous works have considered the impact of reimbursement systems on equity in access to primary care, the quality of primary care [20], and interprofessional collaboration and integrated service delivery in primary and allied health care [12,14,21]. Therefore, by investigating the impact that funding models and policies may have on interprofessional collaboration and integrated health care service delivery at the primary care level, the knowledge gained by this scoping review is intended to address a notable research gap. Further, it will inform future research projects investigating the role of health care funding, interprofessional collaboration, and service integration in improving health care access, efficiency, effectiveness, and sustainability in line with UHC ideals.

### Review Questions

#### Primary Research Question

How do health care funding policies and models impact interprofessional collaboration and integrated service delivery in primary and allied care? 

#### Secondary Research Questions

The secondary research questions are as follows:

1. Which key health care funding models and policies determine health care funding in primary and allied health care?
2. Which key characteristics of interprofessional collaboration and integrated health care have been researched in relation to health care funding in primary and allied care?
3. What impact does funding have on professional roles and responsibilities when working in an integrated primary or allied health care role?

## Methods

### Overview

The proposed scoping review will be conducted in accordance with the JBI Institute methodology for scoping reviews [22-24] and will use the PRISMA-ScR (Preferred Reporting Items for Systematic Reviews and Meta-analyses extension for Scoping Reviews) reporting guidelines and checklist to ensure appropriate methodological rigor and transparency [23].

### Inclusion Criteria

#### Participants

This review will consider literature pertaining to adults (defined as individuals ages 18 years and older) who are employed within the specified primary and allied health care disciplines or are the recipients of care provided by these disciplines. 

For the purposes of this review, primary health care professions will include physicians, physician assistants, nurses, and allied health professionals identified as working in primary health care settings. There is no international consensus on what constitutes allied health or the specific professions and disciplines included within the allied health field [25]. Therefore, the extensive list of potential inclusions makes achieving sufficient data saturation for all nationally and internationally recognized allied health professions impractical within the constraints of this review. As such, allied health professions were selected for inclusion based on the following criteria: (1) being recognized as an allied health profession and represented by a professional association member or affiliate member of Allied Health Professions Australia [26], (2) having a scope of practice that includes interprofessional collaboration and integration at the primary health care level, (3) having a practice that is treatment-based rather than diagnostic, and (4) not being recognized as an alternative health or complementary therapy discipline.

Therefore, the allied health professionals selected for inclusion in this scoping review are audiologists, chiropractors, dietitians, exercise physiologists, hand therapists, myotherapists, occupational therapists, optometrists, orthoptists, orthotists, osteopaths, physiotherapists, podiatrists, prosthetists, psychologists, rehabilitation counselors, social workers, and speech and language therapists. Multimedia Appendix 1 further outlines the rationale for the exclusion of several other allied health professions. Due to feasibility constraints, a decision was made to exclude literature concerning interprofessional collaboration and service integration involving secondary (eg, specialist services such as psychiatry, oncology, or surgery) or tertiary (eg, hospital) levels of care.

#### Concept

The following 3 concepts will be explored in this scoping review: health care funding, interprofessional collaboration, and integrated service delivery. Regarding health care funding, factors of particular interest will include health care funding models (eg, compensable, noncompensable, public, and private funding models) and health care funding policies (eg, governmental, nongovernmental, health system, organizational, or professional funding policies). Health care funding characteristics are also of interest. These will include units of funding (eg, per service, per hour, per case, or per capita funding), funding principles (eg, input-based, output-based, performance-based, or achievement-based funding), the timing of funding (eg, prospective, or retrospective funding), and funding modes (eg, cash, recourses, assets, or in-kind services) [27].

The elements of interprofessional collaboration addressed by this review will focus on the relationships, interactions, and collaborative processes between primary and allied health clinicians (eg, writing and receiving referrals and participating in case conferences, clinical discussions, and multipractitioner appointments, assessments, and interventions) [7,9-12,18]. As for health care integration, the type (eg, normative or functional integration), and level (eg, system, organizational, professional, or clinical integration), of horizontal connections between primary and allied health clinicians will be considered [3,6,8,9].

#### Context

This scoping review will consider health care funding policies and funding models operating at the primary health care level only. The primary level was selected as it has long been stipulated that improving health care funding and implementing policies that facilitate greater interprofessional collaboration and service integration between primary and allied health care at this level could improve the ongoing quality and safety and future sustainability of the wider health care system [2,3,8,14-17].

#### Types of Sources Included in the Search

All classifications of primary studies inclusive of quantitative, qualitative, and mixed-methods designs will be considered for inclusion. In addition, grey literature (including policy documents, government and organizational reports, academic theses and dissertations, white papers, book chapters, conference abstracts and proceedings, policy and procedure documents, and opinion papers) will also be considered. Due to feasibility constraints, the scoping review inclusion criteria will be limited to digitally available contemporary literature accessible in the English language (due to the absence of dedicated funding that would typically cover the cost of translation services). A contemporary period of 10 years (from 2011 to 2021) was selected for this review to capture the increased interest in health care funding models and policies post economic recovery from the global financial crisis between 2007 and 2009 [28].

### Search Strategy

The search strategy will aim to locate published and unpublished literature. An initial limited search of MEDLINE and CINAHL identified relevant articles and the index terms were used to develop a search strategy for MEDLINE (Ovid), with input from a professional research librarian (Multimedia Appendix 2). The search strategy, including all identified keywords and index terms, will be adapted as appropriate for each database and information source. Reference lists of retrieved literature that meet the inclusion criteria will be manually screened to identify additional relevant sources.

A search of published literature will be conducted using the following electronic databases: Ovid (Allied and Complementary Medicine Database, EMBASE, Emcare, MEDLINE, and PsycINFO), EBSCOhost (CINAHL), Scopus, and Web of Science. Sources of unpublished studies and grey literature to be searched will include GreyLit, Google Scholar, Open Access Theses and Dissertations, ProQuest Dissertations & Theses Global, Public Affairs Information Service Index, the WHO, and government health departments of primarily English-speaking countries (eg, Australia, Canada, New Zealand, the Republic of Ireland, South Africa, the United Kingdom, and the United States of America).

#### Source of Evidence Selection

Following search strategy implementation, the literature will be collated and uploaded into EndNote version X9 (Clarivate Analytics) for the removal of duplicate entries. Citations will then be exported to Covidence (Veritas Health Innovation), for screening of the titles and abstracts. Screening will be conducted against the inclusion criteria by 2 independent reviewers, with a third independent reviewer available to resolve any potential disagreements. Literature that the reviewers agree has met the inclusion criteria in the initial round of screening will be retrieved in full text and assessed against the inclusion criteria by the same independent reviewers, noting the reasons for any exclusions. The final search results (including reasons for exclusion) will be reported in full and presented using a PRISMA-ScR flow diagram [24].

#### Data Extraction

A total of 2 independent reviewers will use a custom data extraction tool developed by the authors to extract data from the included studies. Data for extraction (Textbox 1) will include descriptive information about the resource in terms of the author(s), year, country of origin, research aims, design, methodology, and methods. Information about funding model characteristics (including the funding type, level, unit, amount, principle, timing, and mode), funding policy characteristics (including the policy type, level, and scope), and practitioner characteristics (including the sample size and disciplines of health care practitioners), will be included. The research findings and any relevant outcome measures used in the research will also be extracted. This data extraction tool may undergo further modification and refinement during the data extraction process, with any changes to be clearly outlined in the final scoping review.

Although not a requirement of scoping review methodology [22,23], we decided to include an assessment of the level of evidence (using the Research Evidence Appraisal Tool and Non-Research Evidence Appraisal Tool) [29] and methodological quality (using the Crowe Critical Appraisal Tool [CCAT]) [30]. Including this optional step is intended to improve the robustness of the scoping review findings [31] and will allow for a more consistent analysis of what is predicted to be considerable heterogeneity in the literature.

**Sample data extraction elements.**

**Article information**

- Author(s)
- Date
- Country of origin
- Aims or purpose
- Research methodology

**Funding model characteristics**

- Funding type, level, unit, principle, timing, and mode

**Interprofessional collaboration and service integration characteristics**

- Type and purpose of interprofessional collaboration and service integration

**Practitioner characteristics**

- Number and disciplines of primary and allied health care professionals

**Findings**

- Key findings related to how health care funding policies or characteristics impact interprofessional collaboration and service integration

**Level of evidence and quality rating**

- Level of evidence and quality rating will be performed using the Research Evidence Appraisal Tool and Non-Research Evidence Appraisal Tool [29].

**Critical appraisal score**

- Critical appraisal will be performed using the Crowe Critical Appraisal Tool Form (v1.4) [30].

#### Data Analysis and Presentation

Due to the broad nature of the research questions and the descriptive purpose of this scoping review, considerable heterogeneity of the literature is expected. It is anticipated that data presentation and analysis will need to accommodate for heterogeneity in the following factors:

- Research methodology and approaches (ie, qualitative, quantitative, mixed-methods, and nonexperimental literature will be considered, as will academic and grey literature)
- Funding model characteristics (ie, the funding model type, level, and scope)
- Funding policy characteristics (ie, the policy type, level, and scope)
- Interprofessional collaboration and service integration characteristics (ie, the type and purpose for collaboration and the type of service integration)
- Health care practitioner characteristics (ie, a range of primary and allied health care disciplines will be considered)

To meaningfully account for this complexity in the literature and provide a clear and accurate map of the evidence, a descriptive narrative analysis method of translating the research findings is proposed. Based on the 4-element convergent narrative synthesis framework outlined by Popay et al [31], a modified 3-element analysis encompassing (1) preliminary analysis, (2) robustness evaluation, and (3) relationship exploration is proposed.

The preliminary analysis will be conducted at the data extraction stage of the review. It will entail organizing and presenting the extracted data (Textbox 1) in tabular form to identify key themes. Also presented in the data extraction table will be the robustness evaluation. This will critically appraise the methodological quality of the literature using the CCAT (v1.4) [30] and assign a level of evidence to the included literature using the Johns Hopkins Nursing Evidence-Based Practice Evidence Level and Quality Guide Research Evidence Appraisal Tool [29] and Non-Research Evidence Appraisal Tool [29]. The relationship exploration will then consider the relationships and variability between the literature and identified themes, presented as a concept map that systematically highlights the evidence and research gaps that inform health care funding practices, policymaking, and research.

## Results

Electronic database searches were conducted in November 2021, and 8013 results were retrieved. Title and abstract screening, full-text screening, data extraction, and manuscript completion are expected in May 2022. Upon completion of the final manuscript, the scoping review is intended for publication in a peer-reviewed academic journal.

## Discussion

The findings from this review will identify the extent and nature of evidence regarding health care funding and how it impacts interprofessional collaboration and service integration at the primary and allied health care levels. These findings will be used to inform future research projects investigating the role of primary health care funding, interprofessional collaboration, and service integration in improving health care access, efficiency, effectiveness, and sustainability.

## Appendix

Multimedia Appendix 1 Examples of excluded health professions.

Multimedia Appendix 2 Database search strategy.

## Abbreviations

CCAT: Crowe Critical Appraisal Tool
PRISMA-ScR: Preferred Reporting Items for Systematic Reviews and Meta-Analyses extension for Scoping Reviews
UHC: Universal Health Care
WHO: World Health Organization
