# Supplementary material for: The Impact of Health Care Funding on Interprofessional Collaboration and Integrated Service Delivery in Primary and Allied Care: Protocol for a Scoping Review
Source: JMIR Res Protoc. 2022 May 13;11(5):e36448. doi: 10.2196/36448 (PMC9143773; doi:10.2196/36448)
Supplement: Multimedia Appendix 1 [file resprot_v11i5e36448_app1.docx]

## Multimedia Appendix 1: [Examples of Excluded Health Professions]

Table 1. Excluded health professions.

| **Professions** | **Reason for exclusion** |
| --- | --- |
| Genetic counsellors; medical imaging and radiation therapists (e.g., diagnostic radiographers, medical imaging technologists, nuclear medicine scientists, nuclear medicine technologists, radiologists, radiation therapists, sonographers); midwives; paramedics; perfusionists | Often closely associated with secondary, pre-tertiary, or tertiary levels of care, these professions were considered for the purposes of this review to be less likely to be represented in the relevant literature on interprofessional primary healthcare teams. |
| Art therapists; dance and movement therapists; drama therapists; music therapists; play therapists; recreation therapists | As emerging, modality-specific therapies, these professions were considered for the purposes of this review to be less likely to be represented in the relevant literature on interprofessional primary healthcare teams. |
| Naturopaths; herbal medicine practitioners; acupuncturists | Frequently represented as alternative or complementary therapies, these professions were considered for the purposes of this review to be less likely to be represented in the relevant literature on interprofessional primary healthcare teams. |
| Pharmacists | Funding for pharmacist care is frequently tied to medication funding models and policies which are beyond the scope of this review. |
