# Supplementary material for: The Impact of Health Care Funding on Interprofessional Collaboration and Integrated Service Delivery in Primary and Allied Care: Protocol for a Scoping Review
Source: JMIR Res Protoc. 2022 May 13;11(5):e36448. doi: 10.2196/36448 (PMC9143773; doi:10.2196/36448)
Supplement: Multimedia Appendix 2 [file resprot_v11i5e36448_app2.docx]

## Multimedia Appendix 2: ]Database Search Strategy]

AMED allied and complimentary medicine - Search conducted on November 6, 2021.

| **Search** | **Query** | **Records retrieved** |
| --- | --- | --- |
| 1 | (health or health care or healthcare or health-care or health system or healthcare system or health service or healthcare service).mp. [mp=abstract, heading words, title] | 64610 |
| 2 | (funding or fund or fee or fees or financing or payment or budget or billing or compensation or spending or insurance or expenditure or expense or imbursement or reimbursement or costing or cost).mp. [mp=abstract, heading words, title] | 10705 |
| 3 | (((((balance billing or balance payment or balance-billing or balance-payment or billing or budget or budget grants or budget policy or budget* or capital financing or capital spending or capitat* or capitation or capitation billing or capitation fee* or capitation finance or capitation or funding or capitation policy or capitation spending or care plan funding or care-plan funding or co finance or co fund or co fund* or co funded or co funding or co pay or co paymen* or co payment or co-finance or co-financing or co-fund or co-fund* or co-funded or co-funding or co-papment or co-pay or co-paymen* or commission based or commission based funding or commission based pay or commission based payment or commission based payments or commission based paying or commission-based or commission-based funding or commission-based pay or commission-based payment or commission-based payments or commission-based paying or community based health insurance or community-based health insurance or compensable or compensable billing or compensable fee* or compensable fund* or compensable funded or compensable funding or compensably funded or compensation or contribution or copay or copayment or cost sharing or cost-sharing or costin* or costin* or costing or costings or coverage or coverage scheme or expenditure or expens* or expense or fee for service or fee* or fees or fee structure or fee model or financ* or finance or finance model or financial model or financing model or financing or financial aid or financial allocation or financial benefits or financial benefits package or financial benefits packages or financial contribution or financial compensation or financial grant or financial imbursement or financial incentiv* or financial incentivisation or financial incentive or financial incentives or financial model or financial obligations or financial outlay or financial options or financial reimbursement or financial reparation or financial restitution or financial subsidy or financial subsidies or financial subsidisation or financial support or financing policy or financial principle or financial policies or financial principles or fund* or funding or funding aid or funding allocation or funding allocation* or funding benefits or funding benefits package or funding benefits packages or funding contribution or funding compensation or funding grant or funding grants or funding imbursement or funding incentiv* or funding incentive or funding incentives or funding incentivisation or funding model or funding obligation or funding obligations or funding options or funding outlay or funding policy or funding principle or funding principles or funding policies or funding reimbursement or financial reparation or funding restitution or funding subsid* or funding subsidies or funding subsidy or funding subsidisation or government funding or grant* or insurance model or health-insurance or insurance or insurance fee or insurance rate or insurance subsidy or insurance subsidies or insurance subsidisation or insurance reimbursement or insurance imbursement or insurance obligations or insurance policies or insurance model or insurance options or insurance incentives or insurance incentivisation or insurance outlay or insurance contribution or insurance payment or insurance policy or insurance rebate or insurance refund or insurance package or insurance packages or mandatory health insurance or opt-in health insurance or opt-out health insurance or Medicaid or Medicare or mixed provider payment or mixed provider payment system or mixed provider policy or mixed-provider payment system or mixed-provider policy or mixed-provider subsid* or mixed-provider-payment or mode of funding or mode of payment or model of funding or NGO funding or NGO funding support or non-government funding or non-government organisation funding or non-government organisation support or out of pocket or out of pocket fee or out of pocket spending or out-of-pocket or out-of-pocket fee or out-of-pocket spending or pay for performance or pay-for-performance or payment or payment incentiv* or payment incentive or payment incentives or payment incentivisation or payment method or payment modality or payment mode or payment options or payment principle or payment scheme or payment syste* or payment system or payment timing or payment type or per-capita fee or per-capita financing or per-capita funding or performance pay or price or private for profit or private for-profit or private funding or private health insurance or private) not for profit) or private) not for profit health insurance) or private not-for-profit or private not-for-profit health insurance or private payment or private-for-profit or private-not-for-profit or prospective paymen* or prospective payment or prospective payments or public funding or public health insurance or public health-insurance or public payment or purchasing or rebat* or rebate or reimburs* or remunerat* or remunerate or remunerate* or remuneration or resource financing or resource spending or retrospective payment or retrospective payments or service funding or service package funding or service payment or service payment* or service payments or service spending or social support fund* or social support funding or social support paymen* or social support payment or social support payments or spending or spending outlay or subsid* or subsidis* or subsidisation or subsidised or subsidised funding or subsidised funding or subsidised payment* or subsidised suppor* or subsidy or support funding or support package or third party billing or third party compensation or third party health insurance or third party insurance coverage or third party rebate or third party refund or third party restitution or third-party billing or third-party compensation or third-party health insurance or third-party insurance coverage or third-party rebate or third-party refund or third-party restitution or type of billing or type of financing or type of funding or type of payment or universal health care or universal health care budget or universal health care funding or universal health care spending or universal health coverage or universal healthcare or universal healthcare budget or universal healthcare funding or universal healthcare spending or voluntary health insurance or voluntary health-insurance or workforce funding or workforce spending).mp. [mp=abstract, heading words, title] | 23927 |
| 4 | 1 and 2 | 5075 |
| 5 | 1 and 3 | 8292 |
| 6 | (inter discipline or inter disciplinary or inter-discipline or interdisciplinary or inter professional or inter profession or inter-profession or interprofessional or inter-professional or multidiscipline or multi discipline or multi disciplinary or multi-disciplinary or multi-discipline or multidisciplinary or trans discipline or transdisciplinary or trans-discipline or trans-disciplinary).mp. [mp=abstract, heading words, title] | 5289 |
| 7 | (care or care co-ordination or care collaboration or care cooperation or care coordination or care delivery or care integration or co-operation or co-operative care or co-ordinated care or collaboration or collaborative care or comprehensive care or cooperation or cooperative care or coordinated care or horizontal care integration or horizontal integration or horizontal service integration or horizontally integrated care or integrated care or integration or integrative care or practice or service or service delivery or team care or teamwork or vertical care integration or vertical integration or vertically integrated care or vertical service integration).mp. [mp=abstract, heading words, title] | 81253 |
| 8 | 6 and 7 | 3720 |
| 9 | 4 and 8 | 215 |
| 10 | 5 and 8 | 394 |
| 11 | (((((((allied health care practitioners or allied health care occupations or allied health care personnel or allied health care practitio* or allied health care practitioner or allied health care professio* or allied health care profession or allied health care professional or allied health care staff or allied health care workers or allied health occupations or allied health personnel or allied health practitio* or allied health practitioner or allied health practitioners or allied health professio* or allied health profession or allied health professional or allied health staff or allied health workers or allied health-care practitioners or allied health-care occupations or allied health-care personnel or allied health-care practitio* or allied health-care practitioner or allied health-care professio* or allied health-care profession or allied health-care professional or allied health-care staff or allied health-care workers or allied healthcare practitioners or allied healthcare occupations or allied healthcare personnel or allied healthcare practitio* or allied healthcare practitioner or allied healthcare professio* or allied healthcare profession or allied healthcare professional or allied healthcare staff or allied healthcare workers or primary health care occupations or primary health care personnel or primary health care practitio* or primary health care practitioner or primary health care practitioners or primary health care professio* or primary health care profession or primary health care professional or primary health care staff or primary health care workers or primary health occupations or primary health personnel or primary health practitio* or primary health practitioner or primary health practitioners or primary health professio* or primary health profession or primary health professional or primary health staff or primary health workers or primary health-care occupations or primary health-care personnel or primary health-care practitio* or primary health-care practitioner or primary health-care practitioners or primary health-care professio* or primary health-care profession or primary health-care professional or primary health-care staff or primary health-care workers or primary healthcare occupations or primary healthcare personnel or primary healthcare practitio* or primary healthcare practitioner or primary healthcare practitioners or primary healthcare professio* or primary healthcare profession or primary healthcare professional or primary healthcare staff or primary healthcare workers or audiologist or audiologists or audiology or chiropodist or chiropodists or chiropody or chiropractic or chiropractor or chiropractors or dietetics or dietitian or dietitians or doctor or doctors or exercise physiologist or exercise physiologists or exercise physiology or family doctor or family medical physician or family medical practitioner or family medicine or general practice or general practitioner or general practitioners or genetic counselling or genetic counsellor or genetic counsellors or GP or GPs or hand therapist or hand therapists or hand therapy or myotherapist or myotherapists or myotherapy or nurse or nurses or nursing or occupational Therapist or occupational therapists or occupational therapy or optometrist or optometrists or optometry or orthoptics or orthoptist or orthoptists or orthotist or orthotists or osteopath or osteopaths or osteopathy or OT or OTs or physician or physicians or physiotherapist or physiotherapists or physiotherapy or podiatrist or podiatrists or podiatry or primary care doctor or primary care nurse or primary care physician or prosthetist or prosthetists or psychologist or psychologists or psychology or social work or social worker or social workers or speech and language therapist or speech and language therapists or speech and language therapy or speech pathologist or speech pathologist or speech pathology or speech pathology or speech therapist or speech therapy).mp. [mp=abstract, heading words, title] | 1331 |
| 12 | 9 and 11 | 2 |
| 13 | 10 and 11 | 5 |

CINAHL Cumulative Index to Nursing and Allied Health Literature - Search conducted on November 6, 2021.

| **Search** | **Query** | **Records retrieved** |
| --- | --- | --- |
| 1 | (MH "Multidisciplinary Care Team") OR (MH "Shared Services, Health Care") OR (MH "Insurance, Health") OR (MH "Health Care Delivery") OR (MH "Universal Health Care") OR (MH "Primary Health Care") OR (MH "United States Centers for Medicare and Medicaid Services") OR "“health” or “health care” or “healthcare” or “health-care” or “health system” or “healthcare system” or “health service” or “healthcare service”" | 209,859 |
| 2 | (MH "Health Care Costs") OR (MH "Costs and Cost Analysis") OR (MH "Cost Benefit Analysis") OR (MH "Insurance, Health, Reimbursement") OR (MH "Fees and Charges") OR (MH "Capitation Fee") OR (MH "Health Facility Charges") OR (MH "Economic Aspects of Illness") OR (MH "Nursing Costs") OR (MH "Fee for Service Plans") OR "“funding” or “fund” or “fee” or “fees” or “financing” or “payment” or “budget “or “billing” or “compensation” or “spending” or “insurance” or “expenditure” or “expense” or “imbursement” or “reimbursement” or “costing” or “cost”" | 142,111 |
| 3 | S1 AND S2 | 22,817 |
| 4 | (MH "Insurance, Health, Reimbursement") OR (MH "Community-Based Health Insurance") OR (MH "Insurance, Health") OR (MH "Health Benefit Plans, Employee") OR (MH "Health Care Costs") OR (MH "Health Insurance Exchanges") OR (MH "Funding Source") OR (MH "Prospective Payment System") OR (MH "Multidisciplinary Care Team") OR (MH "Shared Services, Health Care") | 631,517 |
| 5 | S1 AND S4 | 99,151 |
| 6 | ("“inter discipline” or “inter disciplinary” or “inter-discipline” or “interdisciplinary” or “inter professional” or “inter profession” or “inter-profession” or “interprofessional” or “inter-professional” or “multidiscipline” or “multi discipline” or “multi disciplinary” or “multi-disciplinary” or “multi-discipline” or “multidisciplinary” or “trans discipline” or “transdisciplinary” or “trans-discipline” or “trans-disciplinary”" | 0 |
| 7 | "“inter discipline” or “inter disciplinary” or “inter-discipline” or “interdisciplinary” or “inter professional” or “inter profession” or “inter-profession” or “interprofessional” or “inter-professional” or “multidiscipline” or “multi discipline” or “multi disciplinary” or “multi-disciplinary” or “multi-discipline” or “multidisciplinary” or “trans discipline” or “transdisciplinary” or “trans-discipline” or “trans-disciplinary”" smart searching | 37 |
| 8 | (MH "Health Care Delivery, Integrated") OR (MH "Shared Services, Health Care") OR "“care” or “care co-ordination” or “care collaboration” or “care cooperation” or “care coordination” or “care delivery” or “care integration” or “co-operation” or “co-operative care” or “co-ordinated care” or “collaboration” or “collaborative care” or “comprehensive care” or “cooperation” or “cooperative care” or “coordinated care” or “horizontal care integration” or “horizontal integration” or “horizontal service integration” or “horizontally integrated care” or “integrated care” or “integration” or “integrative care” or “practice” or “service” or “service delivery” or “team care” or “teamwork” or “vertical care integration” or “vertical integration” or “vertically integrated care” or “vertical service integration”" | 13,467 |
| 9 | S7 AND S8 (0) | 0 |
| 10 | S7 AND S8 smart text | 0 |
| 11 | (MH "Multidisciplinary Care Team") OR (MH "Primary Health Care") OR "“allied health care practitioners” or “allied health care occupations” or “allied health care personnel” or “allied health care practitio*” or “allied health care practitioner” or “allied health care professio*” or “allied health care profession” or “allied health care professional” or “allied health care staff” or “allied health care workers” or “allied health occupations” or “allied health personnel” or “allied health practitio*” or “allied health practitioner” or “allied health practitioners” or “allied health professio*” or “allied health profession” or “allied health professional” or “allied health staff” or “allied health workers” or “allied health-care practitioners” or “allied health-care occupations” or “allied health-care personnel” or “allied health-care practitio*” or “allied health-care practitioner” or “allied health-care professio*” or “allied health-care profession” or “allied health-care professional” or “allied health-care staff” or “allied health-care workers” or “allied healthcare practitioners” or “allied healthcare occupations” or “allied healthcare personnel” or “allied healthcare practitio*” or “allied healthcare practitioner” or “allied healthcare professio*” or “allied healthcare profession” or “allied healthcare professional” or “allied healthcare staff” or “allied healthcare workers” or “primary health care occupations” or “primary health care personnel” or “primary health care practitio*” or “primary health care practitioner” or “primary health care practitioners” or “primary health care professio*” or “primary health care profession” or “primary health care professional” or “primary health care staff” or “primary health care workers” or “primary health occupations” or “primary health personnel” or “primary health practitio*” or “primary health practitioner” or “primary health practitioners” or “primary health professio*” or “primary health profession” or “primary health professional” or “primary health staff” or “primary health workers” or “primary health-care occupations” or “primary health-care personnel” or “primary health-care practitio*” or “primary health-care practitioner” or “primary health-care practitioners” or “primary health-care professio*” or “primary health-care profession” or “primary health-care professional” or “primary health-care staff” or “primary health-care workers” or “primary healthcare occupations” or “primary healthcare personnel” or “primary healthcare practitio*” or “primary healthcare practitioner” or “primary healthcare practitioners” or “primary healthcare professio*” or “primary healthcare profession” or “primary healthcare professional” or “primary healthcare staff” or “primary healthcare workers” or “audiologist” or “audiologists” or “audiology” or “chiropodist” or “chiropodists” or “chiropody” or “chiropractic” or “chiropractor” or “chiropractors” or “dietetics” or “dietitian” or “dietitians” or “doctor” or “doctors” or “exercise physiologist” or “exercise physiologists” or “exercise physiology” or “family doctor” or “family medical physician” or “family medical practitioner” or “family medicine” or “general practice” or “general practitioner” or “general practitioners” or “GP” or “GPs” or “hand therapist” or “hand therapists” or “hand therapy” or “myotherapist” or “myotherapists” or “myotherapy” or “nurse” or “nurses” or “nursing” or “nurse practitioner” or “occupational therapist” or “occupational therapists” or “occupational therapy” or “optometrist” or “optometrists” or “optometry” or “orthoptics” or “orthoptist” or “orthoptists” or “orthotist” or orthotists or “osteopath” or “osteopaths” or “osteopathy” or “OT” or “OTs” or “physician” or “physicians” or “physiotherapist” or “physiotherapists” or “physiotherapy” or “podiatrist” or “podiatrists” or “podiatry” or “primary care doctor” or “primary care nurse” or “primary care physician” or “prosthetist” or “prosthetists” or “psychologist” or “psychologists” or “psychology” or “social work” or “social worker” or “social workers” or “speech and language therapist” or “speech and language therapists” or “speech and language therapy” or “speech pathologist” or “speech pathologist” or “speech pathology” or “speech pathology” or “speech therapist” or “speech therapy”" OR (MH "Health Personnel") | 154,374 |
| 12 | S2 OR S4 | 686,338 |
| 13 | S1 AND S12 | 101,961 |
| 14 | S8 AND S11 | 3,424 |
| 15 | S13 AND S14 | 1,848 |
| 16 | S13 AND S14  **Limiters** - Publication Year: 2011-2021; English Language | 851 |

OVID Embase - Search conducted on November 6, 2021.

| **Search** | **Query** | **Records retrieved** |
| --- | --- | --- |
| 1 | (health or health care or healthcare or health-care or health system or healthcare system or health service or healthcare service).mp. [mp=title, abstract, heading word, drug trade name, original title, device manufacturer, drug manufacturer, device trade name, keyword, floating subheading word, candidate term word | 4703513 |
| 2 | (funding or fund or fee or fees or financing or payment or budget or billing or compensation or spending or insurance or expenditure or expense or imbursement or reimbursement or costing or cost).mp. [mp=title, abstract, heading word, drug trade name, original title, device manufacturer, drug manufacturer, device trade name, keyword, floating subheading word, candidate term word] | 1521927 |
| 3 | (((((balance billing or balance payment or balance-billing or balance-payment or billing or budget or budget grants or budget policy or budget* or capital financing or capital spending or capitat* or capitation or capitation billing or capitation fee* or capitation finance or capitation or funding or capitation policy or capitation spending or care plan funding or care-plan funding or co finance or co fund or co fund* or co funded or co funding or co pay or co paymen* or co payment or co-finance or co-financing or co-fund or co-fund* or co-funded or co-funding or co-papment or co-pay or co-paymen* or commission based or commission based funding or commission based pay or commission based payment or commission based payments or commission based paying or commission-based or commission-based funding or commission-based pay or commission-based payment or commission-based payments or commission-based paying or community based health insurance or community-based health insurance or compensable or compensable billing or compensable fee* or compensable fund* or compensable funded or compensable funding or compensably funded or compensation or contribution or copay or copayment or cost sharing or cost-sharing or costin* or costin* or costing or costings or coverage or coverage scheme or expenditure or expens* or expense or fee for service or fee* or fees or fee structure or fee model or financ* or finance or finance model or financial model or financing model or financing or financial aid or financial allocation or financial benefits or financial benefits package or financial benefits packages or financial contribution or financial compensation or financial grant or financial imbursement or financial incentiv* or financial incentivisation or financial incentive or financial incentives or financial model or financial obligations or financial outlay or financial options or financial reimbursement or financial reparation or financial restitution or financial subsidy or financial subsidies or financial subsidisation or financial support or financing policy or financial principle or financial policies or financial principles or fund* or funding or funding aid or funding allocation or funding allocation* or funding benefits or funding benefits package or funding benefits packages or funding contribution or funding compensation or funding grant or funding grants or funding imbursement or funding incentiv* or funding incentive or funding incentives or funding incentivisation or funding model or funding obligation or funding obligations or funding options or funding outlay or funding policy or funding principle or funding principles or funding policies or funding reimbursement or financial reparation or funding restitution or funding subsid* or funding subsidies or funding subsidy or funding subsidisation or government funding or grant* or insurance model or health-insurance or insurance or insurance fee or insurance rate or insurance subsidy or insurance subsidies or insurance subsidisation or insurance reimbursement or insurance imbursement or insurance obligations or insurance policies or insurance model or insurance options or insurance incentives or insurance incentivisation or insurance outlay or insurance contribution or insurance payment or insurance policy or insurance rebate or insurance refund or insurance package or insurance packages or mandatory health insurance or opt-in health insurance or opt-out health insurance or Medicaid or Medicare or mixed provider payment or mixed provider payment system or mixed provider policy or mixed-provider payment system or mixed-provider policy or mixed-provider subsid* or mixed-provider-payment or mode of funding or mode of payment or model of funding or NGO funding or NGO funding support or non-government funding or non-government organisation funding or non-government organisation support or out of pocket or out of pocket fee or out of pocket spending or out-of-pocket or out-of-pocket fee or out-of-pocket spending or pay for performance or pay-for-performance or payment or payment incentiv* or payment incentive or payment incentives or payment incentivisation or payment method or payment modality or payment mode or payment options or payment principle or payment scheme or payment syste* or payment system or payment timing or payment type or per-capita fee or per-capita financing or per-capita funding or performance pay or price or private for profit or private for-profit or private funding or private health insurance or private) not for profit) or private) not for profit health insurance) or private not-for-profit or private not-for-profit health insurance or private payment or private-for-profit or private-not-for-profit or prospective paymen* or prospective payment or prospective payments or public funding or public health insurance or public health-insurance or public payment or purchasing or rebat* or rebate or reimburs* or remunerat* or remunerate or remunerate* or remuneration or resource financing or resource spending or retrospective payment or retrospective payments or service funding or service package funding or service payment or service payment* or service payments or service spending or social support fund* or social support funding or social support paymen* or social support payment or social support payments or spending or spending outlay or subsid* or subsidis* or subsidisation or subsidised or subsidised funding or subsidised funding or subsidised payment* or subsidised suppor* or subsidy or support funding or support package or third party billing or third party compensation or third party health insurance or third party insurance coverage or third party rebate or third party refund or third party restitution or third-party billing or third-party compensation or third-party health insurance or third-party insurance coverage or third-party rebate or third-party refund or third-party restitution or type of billing or type of financing or type of funding or type of payment or universal health care or universal health care budget or universal health care funding or universal health care spending or universal health coverage or universal healthcare or universal healthcare budget or universal healthcare funding or universal healthcare spending or voluntary health insurance or voluntary health-insurance or workforce funding or workforce spending).mp. [mp=title, abstract, heading word, drug trade name, original title, device manufacturer, drug manufacturer, device trade name, keyword, floating subheading word, candidate term word] | 3060364 |
| 4 | 1 and 2 | 767711 |
| 5 | 1 and 3 | 963433 |
| 6 | (inter discipline or inter disciplinary or inter-discipline or interdisciplinary or inter professional or inter profession or inter-profession or interprofessional or inter-professional or multidiscipline or multi discipline or multi disciplinary or multi-disciplinary or multi-discipline or multidisciplinary or trans discipline or transdisciplinary or trans-discipline or trans-disciplinary).mp. [mp=title, abstract, heading word, drug trade name, original title, device manufacturer, drug manufacturer, device trade name, keyword, floating subheading word, candidate term word] | 256420 |
| 7 | (care or care co-ordination or care collaboration or care cooperation or care coordination or care delivery or care integration or co-operation or co-operative care or co-ordinated care or collaboration or collaborative care or comprehensive care or cooperation or cooperative care or coordinated care or horizontal care integration or horizontal integration or horizontal service integration or horizontally integrated care or integrated care or integration or integrative care or practice or service or service delivery or team care or teamwork or vertical care integration or vertical integration or vertically integrated care or vertical service integration).mp. [mp=title, abstract, heading word, drug trade name, original title, device manufacturer, drug manufacturer, device trade name, keyword, floating subheading word, candidate term word] | 5696180 |
| 8 | 6 and 7 | 165552 |
| 9 | 4 and 8 | 16295 |
| 10 | 5 and 8 | 24134 |
| 11 | (((((((allied health care practitioners or allied health care occupations or allied health care personnel or allied health care practitio* or allied health care practitioner or allied health care professio* or allied health care profession or allied health care professional or allied health care staff or allied health care workers or allied health occupations or allied health personnel or allied health practitio* or allied health practitioner or allied health practitioners or allied health professio* or allied health profession or allied health professional or allied health staff or allied health workers or allied health-care practitioners or allied health-care occupations or allied health-care personnel or allied health-care practitio* or allied health-care practitioner or allied health-care professio* or allied health-care profession or allied health-care professional or allied health-care staff or allied health-care workers or allied healthcare practitioners or allied healthcare occupations or allied healthcare personnel or allied healthcare practitio* or allied healthcare practitioner or allied healthcare professio* or allied healthcare profession or allied healthcare professional or allied healthcare staff or allied healthcare workers or primary health care occupations or primary health care personnel or primary health care practitio* or primary health care practitioner or primary health care practitioners or primary health care professio* or primary health care profession or primary health care professional or primary health care staff or primary health care workers or primary health occupations or primary health personnel or primary health practitio* or primary health practitioner or primary health practitioners or primary health professio* or primary health profession or primary health professional or primary health staff or primary health workers or primary health-care occupations or primary health-care personnel or primary health-care practitio* or primary health-care practitioner or primary health-care practitioners or primary health-care professio* or primary health-care profession or primary health-care professional or primary health-care staff or primary health-care workers or primary healthcare occupations or primary healthcare personnel or primary healthcare practitio* or primary healthcare practitioner or primary healthcare practitioners or primary healthcare professio* or primary healthcare profession or primary healthcare professional or primary healthcare staff or primary healthcare workers or audiologist or audiologists or audiology or chiropodist or chiropodists or chiropody or chiropractic or chiropractor or chiropractors or dietetics or dietitian or dietitians or doctor or doctors or exercise physiologist or exercise physiologists or exercise physiology or family doctor or family medical physician or family medical practitioner or family medicine or general practice or general practitioner or general practitioners or genetic counselling or genetic counsellor or genetic counsellors or GP or GPs or hand therapist or hand therapists or hand therapy or myotherapist or myotherapists or myotherapy or nurse or nurses or nursing or occupational Therapist or occupational therapists or occupational therapy or optometrist or optometrists or optometry or orthoptics or orthoptist or orthoptists or orthotist or orthotists or osteopath or osteopaths or osteopathy or OT or OTs or physician or physicians or physiotherapist or physiotherapists or physiotherapy or podiatrist or podiatrists or podiatry or primary care doctor or primary care nurse or primary care physician or prosthetist or prosthetists or psychologist or psychologists or psychology or social work or social worker or social workers or speech) and language therapist) or speech) and language therapists) or speech) and language therapy) or speech pathologist or speech pathologist or speech pathology or speech pathology or speech therapist or speech therapy).mp. [mp=title, abstract, heading word, drug trade name, original title, device manufacturer, drug manufacturer, device trade name, keyword, floating subheading word, candidate term word] | 20587 |
| 12 | 9 and 11 | 96 |
| 13 | 10 and 11 | 206 |

OVID Emcare - Search conducted on November 6, 2021.

| **Search** | **Query** | **Records retrieved** |
| --- | --- | --- |
| 1 | (health or health care or healthcare or health-care or health system or healthcare system or health service or healthcare service).mp. [mp=title, abstract, heading word, drug trade name, original title, device manufacturer, drug manufacturer, device trade name, keyword, floating subheading word, candidate term word | 1797176 |
| 2 | (funding or fund or fee or fees or financing or payment or budget or billing or compensation or spending or insurance or expenditure or expense or imbursement or reimbursement or costing or cost).mp. [mp=title, abstract, heading word, drug trade name, original title, device manufacturer, drug manufacturer, device trade name, keyword, floating subheading word, candidate term word] | 483989 |
| 3 | (((((balance billing or balance payment or balance-billing or balance-payment or billing or budget or budget grants or budget policy or budget* or capital financing or capital spending or capitat* or capitation or capitation billing or capitation fee* or capitation finance or capitation or funding or capitation policy or capitation spending or care plan funding or care-plan funding or co finance or co fund or co fund* or co funded or co funding or co pay or co paymen* or co payment or co-finance or co-financing or co-fund or co-fund* or co-funded or co-funding or co-papment or co-pay or co-paymen* or commission based or commission based funding or commission based pay or commission based payment or commission based payments or commission based paying or commission-based or commission-based funding or commission-based pay or commission-based payment or commission-based payments or commission-based paying or community based health insurance or community-based health insurance or compensable or compensable billing or compensable fee* or compensable fund* or compensable funded or compensable funding or compensably funded or compensation or contribution or copay or copayment or cost sharing or cost-sharing or costin* or costin* or costing or costings or coverage or coverage scheme or expenditure or expens* or expense or fee for service or fee* or fees or fee structure or fee model or financ* or finance or finance model or financial model or financing model or financing or financial aid or financial allocation or financial benefits or financial benefits package or financial benefits packages or financial contribution or financial compensation or financial grant or financial imbursement or financial incentiv* or financial incentivisation or financial incentive or financial incentives or financial model or financial obligations or financial outlay or financial options or financial reimbursement or financial reparation or financial restitution or financial subsidy or financial subsidies or financial subsidisation or financial support or financing policy or financial principle or financial policies or financial principles or fund* or funding or funding aid or funding allocation or funding allocation* or funding benefits or funding benefits package or funding benefits packages or funding contribution or funding compensation or funding grant or funding grants or funding imbursement or funding incentiv* or funding incentive or funding incentives or funding incentivisation or funding model or funding obligation or funding obligations or funding options or funding outlay or funding policy or funding principle or funding principles or funding policies or funding reimbursement or financial reparation or funding restitution or funding subsid* or funding subsidies or funding subsidy or funding subsidisation or government funding or grant* or insurance model or health-insurance or insurance or insurance fee or insurance rate or insurance subsidy or insurance subsidies or insurance subsidisation or insurance reimbursement or insurance imbursement or insurance obligations or insurance policies or insurance model or insurance options or insurance incentives or insurance incentivisation or insurance outlay or insurance contribution or insurance payment or insurance policy or insurance rebate or insurance refund or insurance package or insurance packages or mandatory health insurance or opt-in health insurance or opt-out health insurance or Medicaid or Medicare or mixed provider payment or mixed provider payment system or mixed provider policy or mixed-provider payment system or mixed-provider policy or mixed-provider subsid* or mixed-provider-payment or mode of funding or mode of payment or model of funding or NGO funding or NGO funding support or non-government funding or non-government organisation funding or non-government organisation support or out of pocket or out of pocket fee or out of pocket spending or out-of-pocket or out-of-pocket fee or out-of-pocket spending or pay for performance or pay-for-performance or payment or payment incentiv* or payment incentive or payment incentives or payment incentivisation or payment method or payment modality or payment mode or payment options or payment principle or payment scheme or payment syste* or payment system or payment timing or payment type or per-capita fee or per-capita financing or per-capita funding or performance pay or price or private for profit or private for-profit or private funding or private health insurance or private) not for profit) or private) not for profit health insurance) or private not-for-profit or private not-for-profit health insurance or private payment or private-for-profit or private-not-for-profit or prospective paymen* or prospective payment or prospective payments or public funding or public health insurance or public health-insurance or public payment or purchasing or rebat* or rebate or reimburs* or remunerat* or remunerate or remunerate* or remuneration or resource financing or resource spending or retrospective payment or retrospective payments or service funding or service package funding or service payment or service payment* or service payments or service spending or social support fund* or social support funding or social support paymen* or social support payment or social support payments or spending or spending outlay or subsid* or subsidis* or subsidisation or subsidised or subsidised funding or subsidised funding or subsidised payment* or subsidised suppor* or subsidy or support funding or support package or third party billing or third party compensation or third party health insurance or third party insurance coverage or third party rebate or third party refund or third party restitution or third-party billing or third-party compensation or third-party health insurance or third-party insurance coverage or third-party rebate or third-party refund or third-party restitution or type of billing or type of financing or type of funding or type of payment or universal health care or universal health care budget or universal health care funding or universal health care spending or universal health coverage or universal healthcare or universal healthcare budget or universal healthcare funding or universal healthcare spending or voluntary health insurance or voluntary health-insurance or workforce funding or workforce spending).mp. [mp=title, abstract, heading word, drug trade name, original title, device manufacturer, drug manufacturer, device trade name, keyword, floating subheading word, candidate term word] | 837899 |
| 4 | 1 and 2 | 304093 |
| 5 | 1 and 3 | 392983 |
| 6 | (inter discipline or inter disciplinary or inter-discipline or interdisciplinary or inter professional or inter profession or inter-profession or interprofessional or inter-professional or multidiscipline or multi discipline or multi disciplinary or multi-disciplinary or multi-discipline or multidisciplinary or trans discipline or transdisciplinary or trans-discipline or trans-disciplinary).mp. [mp=title, abstract, heading word, drug trade name, original title, device manufacturer, drug manufacturer, device trade name, keyword, floating subheading word, candidate term word] | 90763 |
| 7 | (care or care co-ordination or care collaboration or care cooperation or care coordination or care delivery or care integration or co-operation or co-operative care or co-ordinated care or collaboration or collaborative care or comprehensive care or cooperation or cooperative care or coordinated care or horizontal care integration or horizontal integration or horizontal service integration or horizontally integrated care or integrated care or integration or integrative care or practice or service or service delivery or team care or teamwork or vertical care integration or vertical integration or vertically integrated care or vertical service integration).mp. [mp=title, abstract, heading word, drug trade name, original title, device manufacturer, drug manufacturer, device trade name, keyword, floating subheading word, candidate term word] | 2071283 |
| 8 | 6 and 7 | 65397 |
| 9 | 4 and 8 | 6626 |
| 10 | 5 and 8 | 9720 |
| 11 | (((((((allied health care practitioners or allied health care occupations or allied health care personnel or allied health care practitio* or allied health care practitioner or allied health care professio* or allied health care profession or allied health care professional or allied health care staff or allied health care workers or allied health occupations or allied health personnel or allied health practitio* or allied health practitioner or allied health practitioners or allied health professio* or allied health profession or allied health professional or allied health staff or allied health workers or allied health-care practitioners or allied health-care occupations or allied health-care personnel or allied health-care practitio* or allied health-care practitioner or allied health-care professio* or allied health-care profession or allied health-care professional or allied health-care staff or allied health-care workers or allied healthcare practitioners or allied healthcare occupations or allied healthcare personnel or allied healthcare practitio* or allied healthcare practitioner or allied healthcare professio* or allied healthcare profession or allied healthcare professional or allied healthcare staff or allied healthcare workers or primary health care occupations or primary health care personnel or primary health care practitio* or primary health care practitioner or primary health care practitioners or primary health care professio* or primary health care profession or primary health care professional or primary health care staff or primary health care workers or primary health occupations or primary health personnel or primary health practitio* or primary health practitioner or primary health practitioners or primary health professio* or primary health profession or primary health professional or primary health staff or primary health workers or primary health-care occupations or primary health-care personnel or primary health-care practitio* or primary health-care practitioner or primary health-care practitioners or primary health-care professio* or primary health-care profession or primary health-care professional or primary health-care staff or primary health-care workers or primary healthcare occupations or primary healthcare personnel or primary healthcare practitio* or primary healthcare practitioner or primary healthcare practitioners or primary healthcare professio* or primary healthcare profession or primary healthcare professional or primary healthcare staff or primary healthcare workers or audiologist or audiologists or audiology or chiropodist or chiropodists or chiropody or chiropractic or chiropractor or chiropractors or dietetics or dietitian or dietitians or doctor or doctors or exercise physiologist or exercise physiologists or exercise physiology or family doctor or family medical physician or family medical practitioner or family medicine or general practice or general practitioner or general practitioners or genetic counselling or genetic counsellor or genetic counsellors or GP or GPs or hand therapist or hand therapists or hand therapy or myotherapist or myotherapists or myotherapy or nurse or nurses or nursing or occupational Therapist or occupational therapists or occupational therapy or optometrist or optometrists or optometry or orthoptics or orthoptist or orthoptists or orthotist or orthotists or osteopath or osteopaths or osteopathy or OT or OTs or physician or physicians or physiotherapist or physiotherapists or physiotherapy or podiatrist or podiatrists or podiatry or primary care doctor or primary care nurse or primary care physician or prosthetist or prosthetists or psychologist or psychologists or psychology or social work or social worker or social workers or speech) and language therapist) or speech) and language therapists) or speech) and language therapy) or speech pathologist or speech pathologist or speech pathology or speech pathology or speech therapist or speech therapy).mp. [mp=title, abstract, heading word, drug trade name, original title, device manufacturer, drug manufacturer, device trade name, keyword, floating subheading word, candidate term word] | 8847 |
| 12 | 9 and 11 | 35 |
| 13 | 10 and 11 | 79 |

Ovid MEDLINE - Search conducted on November 6, 2021.

| **Search** | **Query** | **Records retrieved** |
| --- | --- | --- |
| 1 | health or health care or healthcare or health-care or health system or healthcare system or health service or healthcare service or exp "Delivery of Health Care, Integrated"/ or exp Health Care Costs/ or exp Health Care Reform/ or exp Health Expenditures/ or exp Healthcare Disparities/ | 3006900 |
| 2 | funding or fund or fee or fees or financing or payment or budget or billing or compensation or spending or insurance or expenditure or expense or imbursement or reimbursement or costing or cost | 851333 |
| 3 | balance billing or balance payment or balance-billing or balance-payment or billing or budget or budget grants or budget policy or budget* or capital financing or capital spending or capitat* or capitation or capitation billing or capitation fee* or capitation finance or capitation or funding or capitation policy or capitation spending or care plan funding or care-plan funding or co finance or co fund or co fund* or co funded or co funding or co pay or co paymen* or co payment or co-finance or co-financing or co-fund or co-fund* or co-funded or co-funding or co-papment or co-pay or co-paymen* or commission based or commission based funding or commission based pay or commission based payment or commission based payments or commission based paying or commission-based or commission-based funding or commission-based pay or commission-based payment or commission-based payments or commission-based paying or community based health insurance or community-based health insurance or compensable or compensable billing or compensable fee* or compensable fund* or compensable funded or compensable funding funded or compensation or contribution or copay or copayment or cost sharing or cost-sharing or costin* or costin* or costing or costings or coverage or coverage scheme or expenditure or expens* or expense or fee for service or fee* or fees or fee structure or fee model or financ* or finance or finance model or financial model or financing model or financing or financial aid or financial allocation or financial benefits or financial benefits package or financial benefits packages or financial contribution or financial compensation or financial grant or financial imbursement or financial incentiv* or financial incentivisation or financial incentive or financial incentives or financial model or financial obligations or financial outlay or financial options or financial reimbursement or financial reparation or financial restitution or financial subsidy or financial subsidies or financial subsidisation or financial support or financing policy or financial principle or financial policies or financial principles or fund* or funding or funding aid or funding allocation or funding allocation* or funding benefits or funding benefits package or funding benefits packages or funding contribution or funding compensation or funding grant or funding grants or funding imbursement or funding incentiv* or funding incentive or funding incentives or funding incentivisation or funding model or funding obligation or funding obligations or funding options or funding outlay or funding policy or funding principle or funding principles or funding policies or funding reimbursement or financial reparation or funding restitution or funding subsid* or funding subsidies or funding subsidy or funding subsidisation or government funding or grant* or insurance model or health-insurance or insurance or insurance fee or insurance rate or insurance subsidy or insurance subsidies or insurance subsidisation or insurance reimbursement or insurance imbursement or insurance obligations or insurance policies or insurance model or insurance options or insurance incentives or insurance incentivisation or insurance outlay or insurance contribution or insurance payment or insurance policy or insurance rebate or insurance refund or insurance package or insurance packages or mandatory health insurance or opt-in health insurance or opt-out health insurance or Medicaid or Medicare or mixed provider payment or mixed provider payment system or mixed provider policy or mixed-provider payment system or mixed-provider policy or mixed-provider subsid* or mixed-provider-payment or mode of funding or mode of payment or model of funding or NGO funding or NGO funding support or non-government funding or non-government organisation funding or non-government organisation support or out of pocket or out of pocket fee or out of pocket spending or out-of-pocket or out-of-pocket fee or out-of-pocket spending or pay for performance or pay-for-performance or payment or payment incentiv* or payment incentive or payment incentives or payment incentivisation or payment method or payment modality or payment mode or payment options or payment principle or payment scheme or payment syste* or payment system or payment timing or payment type or per-capita fee or per-capita financing or per-capita funding or performance pay or price or private for profit or private for-profit or private funding or private health insurance or private-not-for-profit or private-not-for-profit-health-insurance or prospective paymen* or prospective payment or prospective payments or public funding or public health insurance or public health-insurance or public payment or purchasing or rebat* or rebate or reimburs* or remunerat* or remunerate or remunerate* or remuneration or resource financing or resource spending or retrospective payment or retrospective payments or service funding or service package funding or service payment or service payment* or service payments or service spending or social support fund* or social support funding or social support paymen* or social support payment or social support payments or spending or spending outlay or subsid* or subsidis* or subsidisation or subsidised or subsidised funding or subsidised funding or subsidised payment* or subsidised suppor* or subsidy or support funding or support package or third party billing or third party compensation or third party health insurance or third party insurance coverage or third party rebate or third party refund or third party restitution or third-party billing or third-party compensation or third-party health insurance or third-party insurance coverage or third-party rebate or third-party refund or third-party restitution or type of billing or type of financing or type of funding or type of payment or universal health care or universal health care budget or universal health care funding or universal health care spending or universal health coverage or universal healthcare or universal healthcare budget or universal healthcare funding or universal healthcare spending or voluntary health insurance or voluntary health-insurance or workforce funding or workforce spending | 1899885 |
| 4 | 2 or 3 | 2235486 |
| 5 | 1 and 4 | 684795 |
| 6 | inter discipline or inter disciplinary or inter-discipline or interdisciplinary or inter professional or inter profession or inter-profession or interprofessional or inter-professional or multidiscipline or multi discipline or multi disciplinary or multi-disciplinary or multi-discipline or multidisciplinary or trans discipline or transdisciplinary or trans-discipline | 187613 |
| 7 | care or care co-ordination or care collaboration or care cooperation or care coordination or care delivery or care integration or co-operation or co-operative care or co-ordinated care or collaboration or collaborative care or comprehensive care or cooperation or cooperative care or coordinated care or horizontal care integration or horizontal integration or horizontal service integration or horizontally integrated care or integrated care or integration or integrative care or practice or service or service delivery or team care or teamwork or exp Intersectoral Collaboration/ or exp Cooperative Behavior/ or exp "Attitude of Health Personnel"/ or exp "Delivery of Health Care"/ or exp Interdisciplinary Communication/ | 3564737 |
| 8 | 6 and 7 | 125345 |
| 9 | 5 and 8 | 9057 |
| 10 | allied health care practitioners or allied health care occupations or allied health care personnel or allied health care practitio* or allied health care practitioner or allied health care professio* or allied health care profession or allied health care professional or allied health care staff or allied health care workers or allied health occupations or allied health personnel or allied health practitio* or allied health practitioner or allied health practitioners or allied health professio* or allied health profession or allied health professional or allied health staff or allied health workers or allied health-care practitioners or allied health-care occupations or allied health-care personnel or allied health-care practitio* or allied health-care practitioner or allied health-care professio* or allied health-care profession or allied health-care professional or allied health-care staff or allied health-care workers or allied healthcare practitioners or allied healthcare occupations or allied healthcare personnel or allied healthcare practitio* or allied healthcare practitioner or allied healthcare professio* or allied healthcare profession or allied healthcare professional or allied healthcare staff or allied healthcare workers or primary health care occupations or primary health care personnel or primary health care practitio* or primary health care practitioner or primary health care practitioners or primary health care professio* or primary health care profession or primary health care professional or primary health care staff or primary health care workers or primary health occupations or primary health personnel or primary health practitio* or primary health practitioner or primary health practitioners or primary health professio* or primary health profession or primary health professional or primary health staff or primary health workers or primary health-care occupations or primary health-care personnel or primary health-care practitio* or primary health-care practitioner or primary health-care practitioners or primary health-care professio* or primary health-care profession or primary health-care professional or primary health-care staff or primary health-care workers or primary healthcare occupations or primary healthcare personnel or primary healthcare practitio* or primary healthcare practitioner or primary healthcare practitioners or primary healthcare professio* or primary healthcare profession or primary healthcare professional or primary healthcare staff or primary healthcare workers or audiologist or audiologists or audiology or chiropodist or chiropodists or chiropody or chiropractic or chiropractor or chiropractors or dietetics or dietitian or dietitians or doctor or doctors or exercise physiologist or exercise physiologists or exercise physiology or family doctor or family medical physician or family medical practitioner or family medicine or general practice or general practitioner or general practitioners or GP or GPs or hand therapist or hand therapists or hand therapy or myotherapist or myotherapists or myotherapy or nurse or nurses or nursing or occupational therapist or occupational therapists or occupational therapy or optometrist or optometrists or optometry or orthoptics or orthoptist or orthoptists or orthotist or orthotists or osteopath or osteopaths or osteopathy or OT or OTs or physician or physicians or physiotherapist or physiotherapists or physiotherapy or podiatrist or podiatrists or podiatry or primary care doctor or primary care nurse or primary care physician or prosthetist or prosthetists or psychologist or psychologists or psychology or rehab* counsel* or rehabilitation counselling or rehabilitation counsellor or social work or social worker or social workers or speech language therapist or speech language therapists or speech language therapy or speech pathologist or speech pathologist or speech pathology or speech pathology or speech therapist or speech therapy | 2530298 |
| 11 | 9 and 10 | 4670 |
| 12 | limit 15 to (English language and ovid full text available and humans and ("adult (19 to 44 years)" or "middle age (45 to 64 years)" or "middle aged (45 plus years)" or "all aged (65 and over)" or "aged (80 and over)") and last 10 years) | 84 |

ProQuest Dissertations & Theses Global - Search conducted on November 6, 2021.

| **Search** | **Query** | **Records retrieved** |
| --- | --- | --- |
| 1 | (primary healthcare) AND (allied healthcare) AND funding AND (interprofessional collaboration) AND (integrated healthcare) AND la.exact("English") AND (la.exact("ENG") NOT subt.exact("health education" OR "higher education" OR "education" OR "educational leadership" OR "adult education" OR "curriculum development" OR "educational evaluation" OR "educational technology" OR "business administration" OR "pedagogy" OR "information technology" OR "educational administration" OR "educational psychology" OR "higher education administration" OR "obstetrics" OR "community college education" OR "dentistry" OR "public administration" OR "hospitals" OR "oncology" OR "continuing education" OR "medical imaging" OR "teaching" OR "gender studies" OR "instructional design" OR "mortality" OR "philosophy" OR "public health education" OR "pharmaceutical sciences" OR "educational sociology" OR "surgery" OR "covid-19" OR "criminology" OR "educational tests & measurements" OR "palliative care" OR "pharmacology" OR "school counseling" OR "design" OR "early childhood education" OR "entrepreneurship" OR "learning" OR "awards & honors" OR "children & youth" OR "corporate culture" OR "dance") NOT diskw.exact("Interprofessional education" OR "Nursing education" OR "Hospitals" OR "Breastfeeding" OR "Education" OR "End of life") AND pd(20110101-20211231)) | 625 |

PsychINFO - Search conducted on November 6, 2021.

| **Search** | **Query** | **Records retrieved** |
| --- | --- | --- |
| 1 | (health or health care or healthcare or health-care or health system or healthcare system or health service or healthcare service).mp. [mp=title, abstract, heading word, table of contents, key concepts, original title, tests & measures, mesh] | 763709 |
| 2 | (funding or fund or fee or fees or financing or payment or budget or billing or compensation or spending or insurance or expenditure or expense or imbursement or reimbursement or costing or cost).mp. [mp=title, abstract, heading word, table of contents, key concepts, original title, tests & measures, mesh] | 168208 |
| 3 | (((((balance billing or balance payment or balance-billing or balance-payment or billing or budget or budget grants or budget policy or budget* or capital financing or capital spending or capitat* or capitation or capitation billing or capitation fee* or capitation finance or capitation or funding or capitation policy or capitation spending or care plan funding or care-plan funding or co finance or co fund or co fund* or co funded or co funding or co pay or co paymen* or co payment or co-finance or co-financing or co-fund or co-fund* or co-funded or co-funding or co-papment or co-pay or co-paymen* or commission based or commission based funding or commission based pay or commission based payment or commission based payments or commission based paying or commission-based or commission-based funding or commission-based pay or commission-based payment or commission-based payments or commission-based paying or community based health insurance or community-based health insurance or compensable or compensable billing or compensable fee* or compensable fund* or compensable funded or compensable funding or compensably funded or compensation or contribution or copay or copayment or cost sharing or cost-sharing or costin* or costin* or costing or costings or coverage or coverage scheme or expenditure or expens* or expense or fee for service or fee* or fees or fee structure or fee model or financ* or finance or finance model or financial model or financing model or financing or financial aid or financial allocation or financial benefits or financial benefits package or financial benefits packages or financial contribution or financial compensation or financial grant or financial imbursement or financial incentiv* or financial incentivisation or financial incentive or financial incentives or financial model or financial obligations or financial outlay or financial options or financial reimbursement or financial reparation or financial restitution or financial subsidy or financial subsidies or financial subsidisation or financial support or financing policy or financial principle or financial policies or financial principles or fund* or funding or funding aid or funding allocation or funding allocation* or funding benefits or funding benefits package or funding benefits packages or funding contribution or funding compensation or funding grant or funding grants or funding imbursement or funding incentiv* or funding incentive or funding incentives or funding incentivisation or funding model or funding obligation or funding obligations or funding options or funding outlay or funding policy or funding principle or funding principles or funding policies or funding reimbursement or financial reparation or funding restitution or funding subsid* or funding subsidies or funding subsidy or funding subsidisation or government funding or grant* or insurance model or health-insurance or insurance or insurance fee or insurance rate or insurance subsidy or insurance subsidies or insurance subsidisation or insurance reimbursement or insurance imbursement or insurance obligations or insurance policies or insurance model or insurance options or insurance incentives or insurance incentivisation or insurance outlay or insurance contribution or insurance payment or insurance policy or insurance rebate or insurance refund or insurance package or insurance packages or mandatory health insurance or opt-in health insurance or opt-out health insurance or Medicaid or Medicare or mixed provider payment or mixed provider payment system or mixed provider policy or mixed-provider payment system or mixed-provider policy or mixed-provider subsid* or mixed-provider-payment or mode of funding or mode of payment or model of funding or NGO funding or NGO funding support or non-government funding or non-government organisation funding or non-government organisation support or out of pocket or out of pocket fee or out of pocket spending or out-of-pocket or out-of-pocket fee or out-of-pocket spending or pay for performance or pay-for-performance or payment or payment incentiv* or payment incentive or payment incentives or payment incentivisation or payment method or payment modality or payment mode or payment options or payment principle or payment scheme or payment syste* or payment system or payment timing or payment type or per-capita fee or per-capita financing or per-capita funding or performance pay or price or private for profit or private for-profit or private funding or private health insurance or private) not for profit) or private) not for profit health insurance) or private not-for-profit or private not-for-profit health insurance or private payment or private-for-profit or private-not-for-profit or prospective paymen* or prospective payment or prospective payments or public funding or public health insurance or public health-insurance or public payment or purchasing or rebat* or rebate or reimburs* or remunerat* or remunerate or remunerate* or remuneration or resource financing or resource spending or retrospective payment or retrospective payments or service funding or service package funding or service payment or service payment* or service payments or service spending or social support fund* or social support funding or social support paymen* or social support payment or social support payments or spending or spending outlay or subsid* or subsidis* or subsidisation or subsidised or subsidised funding or subsidised funding or subsidised payment* or subsidised suppor* or subsidy or support funding or support package or third party billing or third party compensation or third party health insurance or third party insurance coverage or third party rebate or third party refund or third party restitution or third-party billing or third-party compensation or third-party health insurance or third-party insurance coverage or third-party rebate or third-party refund or third-party restitution or type of billing or type of financing or type of funding or type of payment or universal health care or universal health care budget or universal health care funding or universal health care spending or universal health coverage or universal healthcare or universal healthcare budget or universal healthcare funding or universal healthcare spending or voluntary health insurance or voluntary health-insurance or workforce funding or workforce spending).mp. [mp=title, abstract, heading word, table of contents, key concepts, original title, tests & measures, mesh] | 709226 |
| 4 | 1 and 2 | 65351 |
| 5 | 1 and 3 | 152425 |
| 6 | (inter discipline or inter disciplinary or inter-discipline or interdisciplinary or inter professional or inter profession or inter-profession or interprofessional or inter-professional or multidiscipline or multi discipline or multi disciplinary or multi-disciplinary or multi-discipline or multidisciplinary or trans discipline or transdisciplinary or trans-discipline or trans-disciplinary).mp. [mp=title, abstract, heading word, table of contents, key concepts, original title, tests & measures, mesh] | 57066 |
| 7 | (care or care co-ordination or care collaboration or care cooperation or care coordination or care delivery or care integration or co-operation or co-operative care or co-ordinated care or collaboration or collaborative care or comprehensive care or cooperation or cooperative care or coordinated care or horizontal care integration or horizontal integration or horizontal service integration or horizontally integrated care or integrated care or integration or integrative care or practice or service or service delivery or team care or teamwork or vertical care integration or vertical integration or vertically integrated care or vertical service integration).mp. [mp=title, abstract, heading word, table of contents, key concepts, original title, tests & measures, mesh] | 953701 |
| 8 | 6 and 7 | 30308 |
| 9 | 4 and 8 | 1718 |
| 10 | 5 and 8 | 3899 |
| 11 | (((((((allied health care practitioners or allied health care occupations or allied health care personnel or allied health care practitio* or allied health care practitioner or allied health care professio* or allied health care profession or allied health care professional or allied health care staff or allied health care workers or allied health occupations or allied health personnel or allied health practitio* or allied health practitioner or allied health practitioners or allied health professio* or allied health profession or allied health professional or allied health staff or allied health workers or allied health-care practitioners or allied health-care occupations or allied health-care personnel or allied health-care practitio* or allied health-care practitioner or allied health-care professio* or allied health-care profession or allied health-care professional or allied health-care staff or allied health-care workers or allied healthcare practitioners or allied healthcare occupations or allied healthcare personnel or allied healthcare practitio* or allied healthcare practitioner or allied healthcare professio* or allied healthcare profession or allied healthcare professional or allied healthcare staff or allied healthcare workers or primary health care occupations or primary health care personnel or primary health care practitio* or primary health care practitioner or primary health care practitioners or primary health care professio* or primary health care profession or primary health care professional or primary health care staff or primary health care workers or primary health occupations or primary health personnel or primary health practitio* or primary health practitioner or primary health practitioners or primary health professio* or primary health profession or primary health professional or primary health staff or primary health workers or primary health-care occupations or primary health-care personnel or primary health-care practitio* or primary health-care practitioner or primary health-care practitioners or primary health-care professio* or primary health-care profession or primary health-care professional or primary health-care staff or primary health-care workers or primary healthcare occupations or primary healthcare personnel or primary healthcare practitio* or primary healthcare practitioner or primary healthcare practitioners or primary healthcare professio* or primary healthcare profession or primary healthcare professional or primary healthcare staff or primary healthcare workers or audiologist or audiologists or audiology or chiropodist or chiropodists or chiropody or chiropractic or chiropractor or chiropractors or dietetics or dietitian or dietitians or doctor or doctors or exercise physiologist or exercise physiologists or exercise physiology or family doctor or family medical physician or family medical practitioner or family medicine or general practice or general practitioner or general practitioners or genetic counselling or genetic counsellor or genetic counsellors or GP or GPs or hand therapist or hand therapists or hand therapy or myotherapist or myotherapists or myotherapy or nurse or nurses or nursing or occupational Therapist or occupational therapists or occupational therapy or optometrist or optometrists or optometry or orthoptics or orthoptist or orthoptists or orthotist or orthotists or osteopath or osteopaths or osteopathy or OT or OTs or physician or physicians or physiotherapist or physiotherapists or physiotherapy or podiatrist or podiatrists or podiatry or primary care doctor or primary care nurse or primary care physician or prosthetist or prosthetists or psychologist or psychologists or psychology or social work or social worker or social workers or speech) and language therapist) or speech) and language therapists) or speech) and language therapy) or speech pathologist or speech pathologist or speech pathology or speech pathology or speech therapist or speech therapy).mp. [mp=title, abstract, heading word, table of contents, key concepts, original title, tests & measures, mesh] | 8019 |
| 12 | 9 and 11 | 8 |
| 13 | 10 and 11 | 27 |

Scopus - Search conducted on November 6, 2021.

| **Search** | **Query** | **Records retrieved** |
| --- | --- | --- |
| 1 | (( TITLE-ABS-KEY ( health OR health AND care OR healthcare OR health-care OR health AND system OR healthcare AND system OR health AND service OR healthcare AND service ) AND ( funding OR fund OR fee OR fees OR financing OR payment OR budget OR billing OR compensation OR spending OR insurance OR expenditure OR expense OR reimbursement OR reimbursement OR costing OR cost ) ) AND ( ( inter AND discipline OR inter AND disciplinary OR inter-discipline OR interdisciplinary OR inter AND professional OR inter AND profession OR inter-profession OR interprofessional OR inter-professional OR multidisciplinary OR multi AND discipline OR multi AND disciplinary OR multi-disciplinary OR multi-discipline OR multidisciplinary OR trans AND discipline OR interdisciplinary OR trans-discipline OR trans-disciplinary ) AND ( care OR care AND co-ordination OR care AND collaboration OR care AND cooperation OR care AND coordination OR care AND delivery OR care AND integration OR co-operation OR co-operative AND care OR co-ordinated AND care OR collaboration OR collaborative AND care OR comprehensive AND care OR cooperation OR cooperative AND care OR coordinated AND care OR horizontal AND care AND integration OR horizontal AND integration OR horizontal AND service AND integration OR horizontally AND integrated AND care OR integrated AND care OR integration OR integrative AND care OR practice OR service OR service AND delivery OR team AND care OR teamwork ) ) | 20 |

Web of Science - Search conducted on November 6, 2021.

| **Search** | **Query** | **Records retrieved** |
| --- | --- | --- |
| 1 | health OR health care OR healthcare OR health-care OR health system OR healthcare system OR health service OR healthcare service (All Fields) | [9,614,475](https://www.webofscience.com/wos/woscc/summary/5fa2dbcd-1d4e-4564-bc49-d646d058b535-066b295c/relevance/1) |
| 2 | ALL=(funding OR fund OR fee OR fees OR financing OR payment OR budget OR billing OR compensation OR spending OR insurance OR expenditure OR expense OR imbursement OR reimbursement OR costing OR cost ) | [6,200,191](https://www.webofscience.com/wos/woscc/summary/51bb366a-b92e-4129-bda0-c1ffcea4fdcb-066b9ae8/relevance/1) |
| 3 | ALL=(inter discipline OR inter disciplinary OR inter-discipline OR interdisciplinary OR inter professional OR inter profession OR inter-profession OR interprofessional OR inter-professional OR multidiscipline OR multi discipline OR multi disciplinary OR multi-disciplinary OR multi-discipline OR multidisciplinary OR trans discipline OR transdisciplinary OR trans-discipline OR trans-disciplinary) | [662,592](https://www.webofscience.com/wos/woscc/summary/af92593c-df2b-4306-b573-ad6d1de67f4c-066ba052/relevance/1) |
| 4 | **((#1) AND #2) AND #3** | [40,104](https://www.webofscience.com/wos/woscc/summary/6588d765-4904-4c45-a920-68f4d798e223-067ec098/relevance/1) |
| 5 | **health OR health care OR healthcare OR health-care OR health system OR healthcare system OR health service OR healthcare service (All Fields) and funding OR fund OR fee OR fees OR financing OR payment OR budget OR billing OR compensation OR spending OR insurance OR expenditure OR expense OR imbursement OR reimbursement OR costing OR cost (All Fields) and inter discipline OR inter disciplinary OR inter-discipline OR interdisciplinary OR inter professional OR inter profession OR inter-profession OR interprofessional OR inter-professional OR multidiscipline OR multi discipline OR multi disciplinary OR multi-disciplinary OR multi-discipline OR multidisciplinary (All Fields) and care co-ordination OR care collaboration OR care cooperation OR care coordination OR care delivery OR care integration OR co-operation OR co-operative care OR co-ordinated care OR collaboration OR collaborative care OR cooperation OR cooperative care OR coordinated care OR horizontal care integration OR horizontal integration OR horizontal service integration OR horizontally integrated care OR integrated care OR integration OR integrative care OR team care OR teamwork(All Fields) and 2011 or 2012 or 2013 or 2014 or 2015 or 2016or 2017 or 2018 or 2019 or 2020 or 2021 (Publication Years)and Theater or Mining Mineral Processing or Ethnic Studiesor Cultural Studies or Crystallography or Nuclear Science Technology or Music or Metallurgy Metallurgical Engineering or Mechanics or Literature or International Relations or History or Entomology or Archaeology or Physical Geography or Philosophy or Mycology or Geochemistry Geophysics or Robotics or Mathematical Methods In Social Sciences or Legal Medicine or Forestry or Demography or Automation Control Systems or Art or Anatomy Morphology or Oceanography or History Philosophy Of Science or Fisheries or Communication or Area Studies or Acoustics or Religion or Imaging Science Photographic Technology or Remote Sensing or Development Studies or Construction Building Technologyor Arts Humanities Other Topics or Electrochemistry or Architecture or Zoology or Transportation or Evolutionary Biology or Polymer Science or Geography or Energy Fuels or Cell Biology or Otorhinolaryngology or Information Science Library Science or Physics or Mathematical Computational Biology or Tropical Medicine or Parasitology or Agricultureor Veterinary Sciences or Meteorology Atmospheric Sciences or Water Resources or Biophysics or Toxicology or Physiology or Instruments Instrumentation or Pathology or Biodiversity Conservation or Food Science Technology or Reproductive Biology or Virology or Anthropology or Sociology or Mathematics or Medical Laboratory Technology or Plant Sciences or Linguistics or Operations Research Management Science or Optics or Urban Studiesor Astronomy Astrophysics or Marine Freshwater Biology or Criminology Penology or Telecommunications or Women S Studies or Geology or Developmental Biology or Biotechnology Applied Microbiology or Microbiology or Materials Science or Emergency Medicine or Dentistry Oral Surgery Medicine or Anesthesiology or Radiology Nuclear Medicine Medical Imaging or Chemistry or Urology Nephrology or Oncology or Science Technology Other Topics or Education Educational Research or Pharmacology Pharmacy or Psychiatry or Pediatrics or Surgery or Environmental Sciences Ecology or Research Experimental Medicine or Engineering or Obstetrics Gynecology or Computer Science or Genetics Heredity or Respiratory System or Biochemistry Molecular Biology or Endocrinology Metabolism or Cardiovascular System Cardiology or Infectious Diseases or Gastroenterology Hepatology or Immunology or Business Economics or Hematology or Rheumatology or Dermatology (Exclude – Research Areas) and English (Languages) and Allergy or Transplantation or Neurosciences Neurology (Exclude – Research Areas) and Substance Abuse (Exclude – Research Areas)** | 4,966 |
